# Structural and functional impact of non-synonymous SNPs in the CST complex subunit TEN1: structural genomics approach

**DOI:** 10.1042/BSR20190312

**Published:** 2019-05-15

**Authors:** Mohd. Amir, Vijay Kumar, Taj Mohammad, Ravins Dohare, Md. Tabish Rehman, Mohamed F. Alajmi, Afzal Hussain, Faizan Ahmad, Md. Imtaiyaz Hassan

**Affiliations:** 1 Centre for Interdisciplinary Research in Basic Sciences, Jamia Millia Islamia, Jamia Nagar, New Delhi 110025, India; 2 Amity Institute of Neuropsychology & Neurosciences, Amity University, Noida, UP 201303, India; 3 Department of Pharmacognosy College of Pharmacy, King Saud University, Riyadh 11451, K.S.A.

**Keywords:** OB fold protein, Pathogenic mutations, Structural genomics, SNP, Sequence analysis

## Abstract

TEN1 protein is a key component of CST complex, implicated in maintaining the telomere homeostasis, and provides stability to the eukaryotic genome. Mutations in *TEN1* gene have higher chances of deleterious impact; thus, interpreting the number of mutations and their consequential impact on the structure, stability, and function is essentially important. Here, we have investigated the structural and functional consequences of nsSNPs in the *TEN1* gene. A wide array of sequence- and structure-based computational prediction tools were employed to identify the effects of 78 nsSNPs on the structure and function of TEN1 protein and to identify the deleterious nsSNPs. These deleterious or destabilizing nsSNPs are scattered throughout the structure of TEN1. However, major mutations were observed in the α1-helix (12–16 residues) and β5-strand (88–96 residues). We further observed that mutations at the C-terminal region were having higher tendency to form aggregate. In-depth structural analysis of these mutations reveals that the pathogenicity of these mutations are driven mainly through larger structural changes because of alterations in non-covalent interactions. This work provides a blueprint to pinpoint the possible consequences of pathogenic mutations in the CST complex subunit TEN1.

## Introduction

Telomeres consist of non-coding ends of eukaryotic linear chromosomes and play a vital role in the replication, regulation, and protection of genome [1,2]. Ends of eukaryotic chromosomes can be identified by recombination and repair system of the cells as DNA strand breaks that often proceed to end-to-end fusion and instability of genome [3,4]. Shelterin complex is composed of six subunits (TRF1, TRF2, RAP1, TIN2, TPP1, and POT1) which are located primarily to single- and double-stranded telomeric DNA [5]. In addition to repressing DDR and chromosome fusion, shelterin complex also caps the telomeric ends by facilitating the formation of T-loop. It is also acting as a processivity factor via recruiting telomerase to chromosomes end [6,7].

The CST complex is composed of three subunits, conserved telomere maintenance component 1 (CTC1), suppressor of CDC thirteen homolog (STN1), and telomere length regulation protein TEN1 homolog (TEN1) [8], specifically localizes to the ssDNA) of telomere and is involved in telomere capping and regulation of telomere length [9–11]. However, increasing evidence has demonstrated that the STN1-TEN1 complex possesses some extra telomeric functions. It is involved in resolving replication fork stalling during replication stress [12,13]. CST complex is also involved in the removal of G-quadruplexes (G4: G-rich repeats) [14]. The G-rich region of the telomere is very prone to form G4 throughout telomeric DNA and poses severe challenges for telomere replication machinery [15]. In addition, CST complex binds to the 3′ ends of telomeres and regulates polymerase α-mediated syntheses of C-strand [16]. Some important reports on the structure and function of the CST complex can be found elsewhere [17–20].

In addition to polymerase α-mediated syntheses of C-strand, a subunit of CST (CTC1-STN1) regulates telomerase-mediated extension of G-rich overhang which is critical for the cell proliferation. Deficiency of CTC1-STN1 complex leads to overextension of G-rich overhangs which initiate DDR [21,22]. In this process, the role of TEN1 is indispensable as it is essential to provide stability to CTC1-STN1 complex. Disruption of TEN1 results in progressive shortening of telomere more like caused by telomerase deficiency. As telomere maintenance is paramount to genome stability, mutations in the genes encoding essential components of CST are associated with varieties of genetic abnormalities including cancer [23], coat plus [24–26], and dyskeratosis congenita [27,28].

Prediction of nsSNPs affecting protein structure and function in detail may be investigated by the aid of cutting-edge computational methods. In many cases, nsSNPs have little or no effect on protein structure and functions, but often a single mutation is highly lethal [29]. Experimental studies suggested that about one-third of nsSNPs are deleterious to human health [30]. Thus, identification of such deleterious nsSNPs is of serious concern in terms of diagnosis and therapeutic perspective. *In vitro* mutational studies by Bryan et al., suggested that mutation in some important residue in *TEN1* gene directly affect the interaction with STN1 to many fold. During structure analysis, we have observed that mutant R27Q caused a marked reduction in the polar interactions between TEN1-STN1complex (Supplementary Figure S1). Disruption in TEN1-STN1 interaction leads to the development telomere malfunctions and thus telomeropathies [31]. A little report is available on the mutational analysis of nsSNPs in *TEN1* gene. Taking this opportunity into consideration and the fact that TEN1 plays crucial role in the telomere maintenance; we have predicted the structural and functional effects of about 78 nsSNPs in the coding region of *TEN1* gene. The present study will offer in-depth understanding of the role of nsSNPs on the structure and function of TEN1 protein.

## Materials and methods

### Data collection

Distribution of nsSNPs in human* TEN1* gene was retrieved from dbSNP [32], Ensembl [33], and HGMD [34] databases. Data enrichment was carried out by removing the variant duplicates of different databases. The human TEN1 amino acid sequence was obtained in FASTA format from UniProt database (UniProt ID: Q86WV5) (http://www.uniprot.org/). A 3D structure of TEN1 (PDB ID: 4JOI) was downloaded from the Protein Data Bank (PDB) [35]. Functional annotations of all SNPs were extracted from the dbSNP database; for example, whether the SNPs present in an intron or exon, in the 3′ or 5′-UTR, or downstream or upstream of the *TEN1* gene.

### Sequence-based prediction of deleterious nsSNPs

Sorting Intolerant from Tolerant (SIFT) (http://sift.jcvi.org/) algorithm was used to predict the amino acid substitution as tolerable and intolerable depending upon the physical and sequence-homology features. Substitutions with normalized probabilities of ≥0.05 and ≤0.05 were predicted as tolerated and deleterious, respectively [36,37]. There were about 78 nsSNPs identified from Ensembl and dbSNP databases. Prediction of tolerated and deleterious effect of these nsSNPs in human *TEN1* gene was predicted using SIFT. Protein variation effect analyzer (PROVEAN) (http://provean.jcvi.org/) tool was used to predict the consequences of amino acid substitution on protein function [38]. It predicts nsSNPs as ‘deleterious’ if the score is less than the threshold value (cutoff is −2.5), and ‘neutral’ if the predicted score is more than the cut-off value. All the nsSNPs in human *TEN1* gene were calculated and analyzed using this cut-off value.

PolyPhen-2 (polymorphism phenotyping-2) (http://genetics.bwh.harvard.edu/pph2/) was used to calculate functional predictions of coding variants. It uses a particular empirical rule comprises of both comparative and physical considerations to predict the probable functional impacts of mutation on the structure–function relationship. FASTA format of protein sequence was used as input to calculate the effects of a particular substitution [39]. It calculates a position-specific independent count (PSIC) score for each substitution and then estimates the score deviations. A mutation is considered as possibly destructive mutation if the PSIC score is ≥0.9.

### Structure-based prediction of destabilizing nsSNPs

STRUM (https://zhanglab.ccmb.med.umich.edu/STRUM/) tool was used to predict the stability differences between WT and mutant proteins. Initially, from protein sequences, a 3D model was generated by I-TASSER simulation and used to train STRUM model through gradient boosting regression. STRUM predicts the possible effects of nsSNPs on the structure and function of a protein using conservation score from an alignment of the multiple-threading template. The query sequence used as input in FASTA format and calculated the impact of a particular substitution in a given sequence [40]. SDM2 (site direct mutator 2) (http://structure.bioc.cam.ac.uk/sdm2) is a knowledge-based tool used to estimate the impact of mutations on the stability of protein [41]. It uses constrained environment-specific substitution tables (ESSTs) to calculate the differences in the protein stability upon mutation [41,42]. SDM2 uses PDB as an input file, and point variants to estimate the stability difference score between the WT and mutants.

PoPMuSiC (http://babylone.ulb.ac.be/popmusic/) tool was used to predict changes in thermodynamic stability upon mutation. PoPMuSiC employing a linear combination of statistical potentials whose coefficients depend on the solvent accessibility of the substituted residues. It uses PDB as an input file. DUET server was used to predict the impact of mutations on the stability of TEN1 protein using PDB code. DUET calculates a combined or consensus predictions of SDM and mCSM (mutation Cutoff Scanning Matrix) using support vector machines (SVMs) in a non-linear regression fashion. The output it provides is in the form of change in Gibbs free energy (∆∆*G*), where negative sign indicates destabilizing mutation [43]. MCSM was implicated to predict the impact of mutations on the stability of proteins using graph-based structural signatures. It predicts protein–protein and protein–nucleic acid interaction [44].

### Identification of diseased phenotype

MutPred (http://mutpred.mutdb.org/) tool was used to predict the association of nsSNPs with disease phenotype [45]. It employs several attributes associated with structure, function, and evolution using PSI-BLAST [46], SIFT [36], and Pfam profiles [47] together with structure disorder prediction tools such as TMHMM [48], DisProt [49], and MARCOIL [50]. Score with g-value more than 0.75 and a p-value less than 0.05 is considered as a confident hypothesis. PhD-SNP, (http://snps.biofold.org/phd-snp/phd-snp.html) is online SVM based prediction tool, was used to predict the pathological effects of a given mutation [51].

### Aggregation propensity analysis

SODA (protein solubility from disorder and aggregation propensity) was used to predict the change in protein solubility upon mutation by comparing the sequence profile of WT and mutants. The aggregation or intrinsic disorder score obtained from PASTA [52], and ESpritz [53], and a combined result obtained from Kyte-Doolittle [54] and FELLS [55]. SODA also predicts types of variation, including insertion and deletion in a given sequence [56].

### Sequence conservation analysis

The importance of a particular amino acid in the structure and functions of protein can be generally retrieved from its conservation score using multiple sequence alignment. The blueprint of amino acid conservation was identified by ConSurf tool, which measures the degree of conservation of each amino acid at a particular position along with the evolutionary profile of amino acid sequence [57]. Conservation score ranged from 1 to 9, where 1 depicts rapidly evolving (variable), 5 indicates region which is evolving moderately, and 9 shows slowly evolving (evolutionarily conserved) position. Exposed residues with high conservation score are being considered as functional whereas buried residues with high conservation score are believed as structural residues.

### Analysis of solvent accessibility

Relative side-chain solvent accessibility (RSA), residue depth and residue-occluded packing density (OSP) of WT, and mutant TEN1 protein have been performed using SDM2 server [41]. It uses ESSTs table to calculate the differences in their RSA, residue depth, and OSP of WT and mutant proteins. RSA has been calculated based on Lee and Richards method [58]. Three classes of relative RSA were defined based on the method of Lee and Richards, whereby a probe of given radius is rolled around the surface of the molecule [58].

## Results and discussion

**Figure 1:**
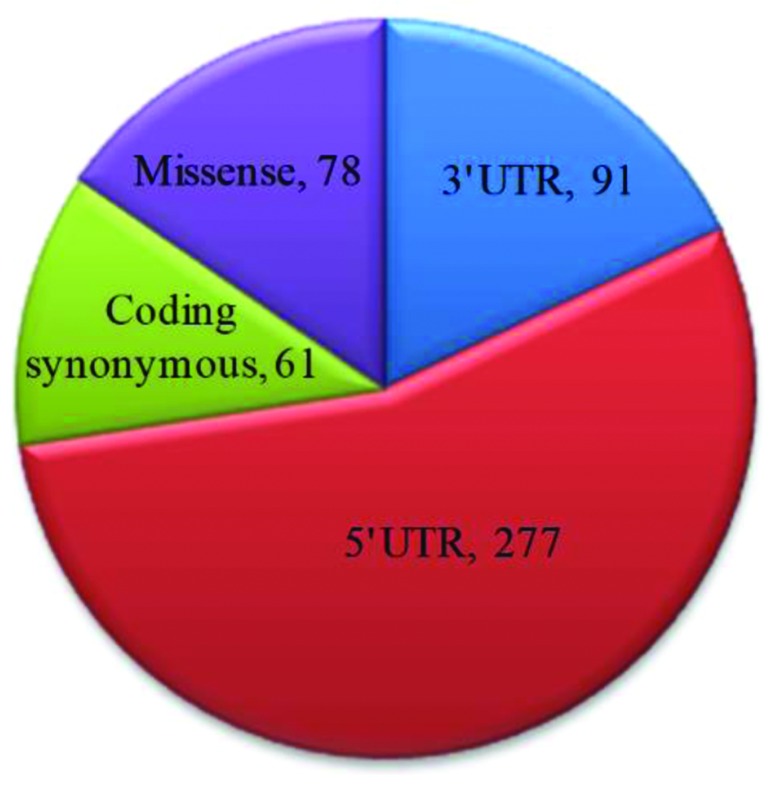
Representation of number of SNPs in *TEN1* gene using dbSNP database

All reported SNPs of *TEN1* gene was extracted from Ensembl (http://www.ensembl.org/) and dbSNP databases (http://www.ncbi.nlm.nih.gov/snp). A total of about 5712 SNPs were mapped and classified into nine different functional classes. Four major classes of SNPs in *TEN1* gene are shown in Figure 1. About 5250 SNPs were mapped in the intronic region and approximately 78 were found in the coding non-synonymous/missense region. The 5′- and 3′-UTR regions have 277 and 91 SNPs, respectively. In addition, 61 SNPs in coding synonymous, five SNPs in frameshift, and three SNPs in each 3′ and 5′ splice site regions are also observed. The present study focuses only on missense mutations mapped in the coding region. A total of 78 nsSNPs were taken for further analysis.

**Figure 2:**
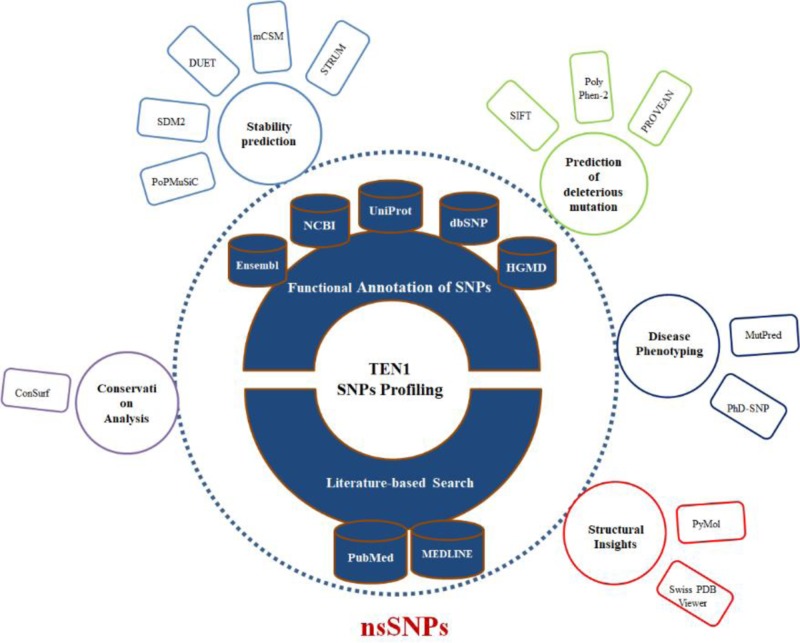
Overview of computational approaches used to identify the deleterious or pathogenic mutations in the TEN1 protein at structural and functional level

To identify the structural and functional impact on missense mutations in *TEN1* gene, we have employed a multi-tier approach. To collect high confidence nsSNPs in the *TEN1* gene, all mapped TEN1 nsSNPs were first subjected to sequence-based prediction using PolyPhen-2, PROVEAN and SIFT, followed by structure-based stability predictions using PoPMuSiC, SDM2, DUET, mCSM, and STRUM web-servers. Further, distributions of high confidence nsSNPs were analyzed on the basis of their structure descriptors and phenotypic association. In consistence, we discuss pathogenic mutations in relation to their sequence conservation, functional importance, and aggregation propensities. Finally, we expand our analysis and extensively analyzed the structural and functional impact of pathogenic mutations on the local environment of the TEN1 protein. An overview of computational methods used in the present study is depicted in Figure 2.

### Identification of deleterious nsSNPs

To pinpoint the structural and functional consequences of nsSNPs in *TEN1* gene, we have performed an extensive structural analysis. The reason for using multiple tools is to improve the confidence level of prediction. Accumulation of deleterious nsSNPs using a single approach may not always be satisfactory as some mutations that have scored very close to cut-off value are prone to false prediction. Therefore, using multiple tools in both sequence- and structure-based predictions may provide an accurate result. The nsSNPs predicted to be deleterious in at least two methods from sequence-based prediction methods and three tools depict destabilizing effects from structure-based prediction were collected and termed as ‘high confidence nsSNPs’.

**Figure 3:**
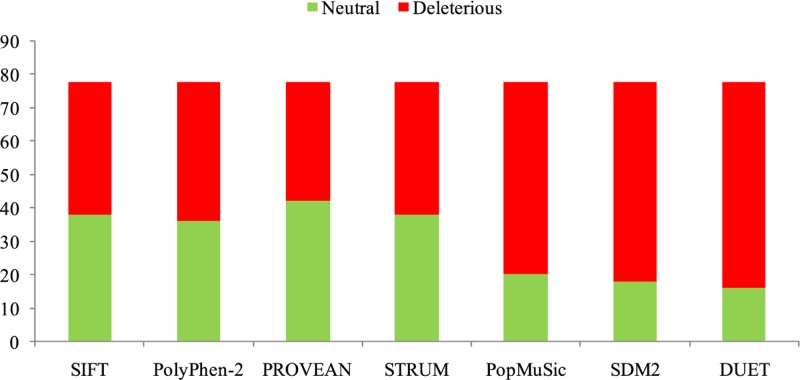
Distribution of predicted deleterious (red) and neutral (green) nsSNPs in *TEN1* gene

Sequence-based prediction of all nsSNPs in *TEN1* gene was calculated by SIFT, PROVEAN, and PolyPhen-2. A total of 78 nsSNPs of human *TEN1* gene were considered for analysis. Sequence**-**and structure-based predictions are listed in Supplementary Table S1 and 2. SIFT, PolyPhen-2, and PROVEAN predicted that out of 78 nsSNPs, 40 (51%), 42 (53%), 36 (46%) nsSNPs, respectively, were deleterious (Figure 3). Similarly, STRUM, mCSM, DUET, SDM2, and PoPMuSiC predicted that 40 (51%), 70 (89%), 62 (79%), 60 (76%), and 58 (74%) nsSNPs, respectively, as protein destabilizing (Figure 3). We have further focussed only on those mutations which are predicted to be deleterious and identified 34 mutations showing a destabilizing behavior.

### Sequence conservation analysis

**Figure 4:**
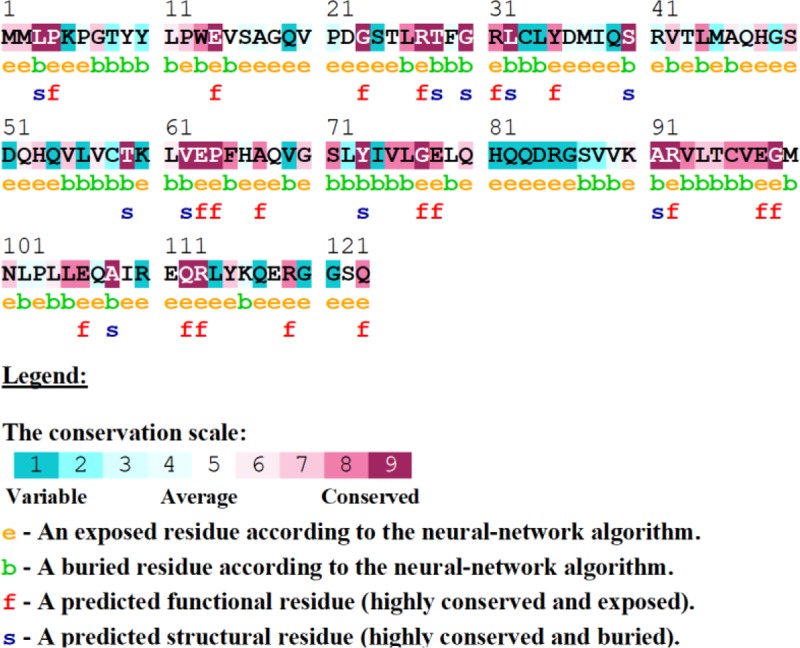
Conservation analysis of the TEN1 protein using ConSurf ConSurf analysis also entails structural importance of a particular residue along with conservation score.

**Figure 5:**
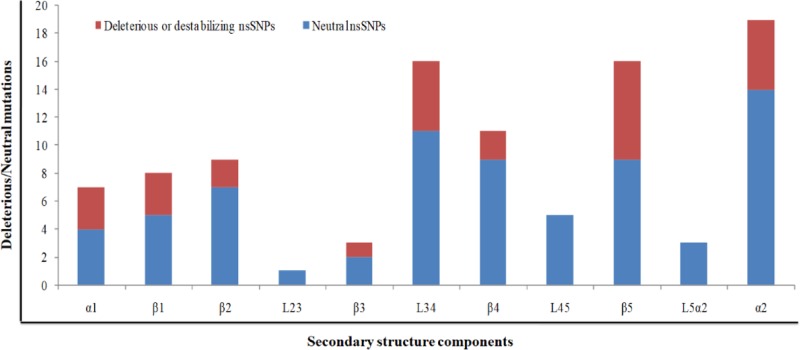
Distribution of deleterious/destabilizing and neutral nsSNPs in different structural components in TEN1 protein

A relative analysis of amino acid residue conservation based on protein sequence provides an understanding of the significance of particular amino acid residue and reveals its localized evolution. ConSurf results indicate that the amino acid residues stretch ranges, 26–32, 62–65, 75–78, and 91–99, were highly conserved (Figure 4). The stretches of amino acids residues range, 32–61 and 100–121, are not conserved. Further, structure-based conservation analysis suggested that amino acid residue belongs to β1 (25–36 residues) and L1-2 (37–40 residues) (loop connecting β1 and β2), β4 (72–80 residues) and β5 (88–96 residues) are more conserved than β2 (41–48 residues) and β3 (51–58 residues) of TEN1 protein. Amongst these structural components, β5 (88-96 residues) is highly conserved while L4-5 (81–87 residues) (loop connecting β4 and β5) is the least conserved. Figure 5.

### Distribution of deleterious or destabilizing nsSNPs

**Figure 6:**
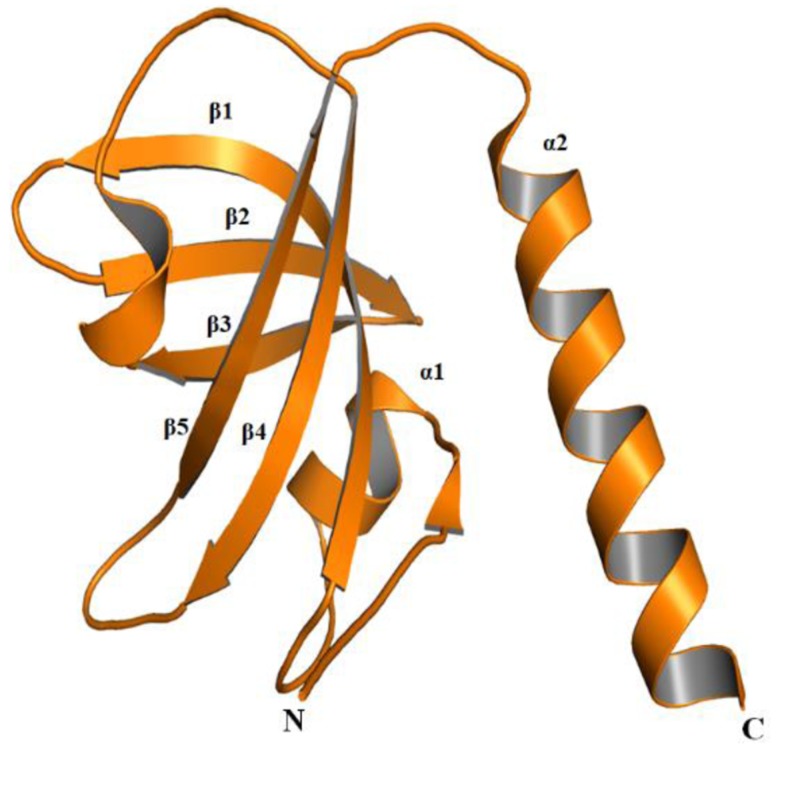
Cartoon representation of TEN1 protein (PDB ID: 4JOI)

TEN1 comprises of 123 amino acid residues and have one OB (oligonucleotide or oligosaccharides)-fold domain (Figure 6) [31]. The OB-folds domain was originally identified from a group of yeast and bacteria [59]. The OB-fold domain can bind and establish protein-DNA, protein-RNA, and protein–protein interactions [60,61]. Amongst these functions, the interaction of OB-folds with ssDNA is extensively studied and characterized [13,62]. Structurally, the OB-folds are β-barrel consisting of five antiparallel β-strands capped by one α-helix at one end has a binding cleft on the other end. The variability in length amongst OB-folds domain is mainly due to the differences in the lengths of variable loops connecting the conserved secondary structure elements [62].

Identification of relative percentage of high confidence nsSNPs in the OB-fold of TEN1 protein provides information about the relationship of a particular secondary structure component to be neutral or pathogenic. The secondary structure components; α1, β1, β2, β3, L3-4 (loop connecting the β3 and β4), β4, β5, α2, respectively have 75, 60, 28, 50, 45, 22, 77, and 35% deleterious or destabilizing mutations (Figure 6). Mutations in the α1 and β5 are having more than 75% chance to be deleterious, while β1, β3, and L3-4 have about 50% chance. In addition, mutations in L1-2, L2-3, L4-5, and L5-α2 (loop connecting β5 and α2) suggested that nsSNPs occurring in these region have negligible chance to be deleterious. From these results, we can suggest that mutations in the α1, β1, and β5 are possibly more lethal than in other parts of TEN1. These observations were further complemented by sequence conservation analysis, which suggested that residues belonging to α1, β1, and β5 of TEN1 are highly conserved.

### Evaluation of disease phenotype

High confidence nsSNPs (deleterious and destabilizing) were analyzed for their phenotypic association using MutPred and PhD-SNP methods (Table 1). These methods predict a particular mutation as benign or pathogenic based on prediction score. MutPred and PhD-SNP methods depict 14 (58%) and 10 (29%), respectively mutations are associated with the disease phenotype. Of the 34 high confidence nsSNPs, we have identified only eight (24%) mutations (W13G, L26P, C58Y, G70A, G77R, R92H, R92C, and C96Y) as pathogenic from both prediction methods. We can conclude that eight (10%) of the total mutation, 78 (100%), found in *TEN1* gene are pathogenic in nature.

**Table 1:** Prediction of disease phenotype analysis of high confidence nsSNPs in *TEN1* gene using PhD-SNP and MutPred prediction tools

S. No.	Variant ID	Variants	PhD-SNP	MutPred2	
			Remark	Score	Remark
1.	rs1322628164	M2V	Neutral	0.329	Benign
2.	rs892524367	P4L	Neutral	0.543	Pathogenic
3.	rs1212831970	Y9C	Disease	0.326	Benign
4.	rs1333358260	W13G	Disease	0.684	Pathogenic
5.	rs1224481693	E14D	Neutral	0.528	Pathogenic
6.	rs1175908725	V15 F	Neutral	0.584	Pathogenic
7.	rs1328038606	G18V	Disease	0.325	Benign
8.	rs964588646	G23E	Neutral	0.744	Pathogenic
9.	rs376979590	T25M	Neutral	0.171	Benign
10.	rs1262136645	L26P	Disease	0.855	Pathogenic
11.	rs1223059981	D36N	Neutral	0.301	Benign
12.	rs1250997925	R41S	Neutral	0.221	Benign
13.	rs1178755431	L44V	Neutral	0.286	Benign
14.	rs1412009927	C58Y	Disease	0.581	Pathogenic
15.	rs977512123	L61M	Neutral	0.168	Benign
16.	rs1032051988	L61W	Neutral	0.575	Pathogenic
17.	rs889310547	P64T	Neutral	0.510	Pathogenic
18.	rs951187486	G70A	Disease	0.482	Pathogenic
19.	rs1180274799	G70S	Neutral	0.545	Pathogenic
20.	rs1445270614	Y73C	Neutral	0.901	Pathogenic
21.	rs1358892195	G77R	Disease	0.880	Pathogenic
22.	rs562062613	V88G	Neutral	0.488	Benign
23.	rs1401886733	A91V	Neutral	0.831	Pathogenic
24.	rs1016457057	R92H	Disease	0.831	Pathogenic
25.	rs759839415	R92C	Disease	0.909	Pathogenic
26.	rs905216603	V93M	Neutral	0.543	Pathogenic
27.	rs1286634889	C96Y	Disease	0.922	Pathogenic
28.	rs1286634889	C96F	Neutral	0.906	Pathogenic
29.	rs368827427	V97M	Neutral	0.707	Pathogenic
30.	rs1216398771	E106D	Neutral	0.226	Benign
31.	rs1230794805	R110W	Neutral	0.130	Benign
32.	rs1158635929	E111G	Neutral	0.280	Benign
33.	rs772974788	R119G	Neutral	0.382	Benign
34.	rs772974788	R119W	Neutral	0.277	Benign

### Analysis of conformational changes in protein structure

**Figure 7:**
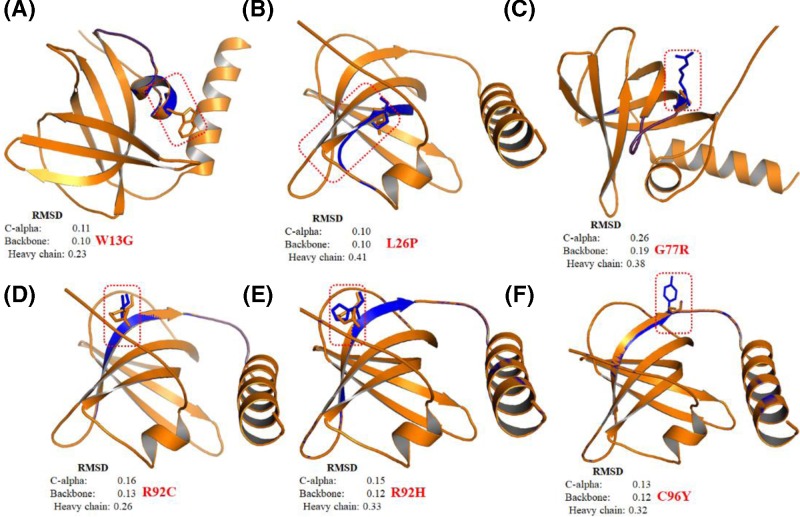
Structural superimposition of Wild-type (Tan color) and mutant (Blue color) TEN1 proteins using PyMol **(A)** W13G, (**B)** L26P, **(C)** G77R, **(D)** R92C, **(E)** R92H, and **(F)** C96Y.

Root mean square deviation (RMSD) is a commonly used quantitative measure of the similarity between two superimposed atomic coordinates, considered as a relative measure of structural and conformational changes in a given protein structure [63]. We have performed a comparative analysis of modeled tertiary structure of mutant proteins with the WT to deduce possible structural and functional consequences imposed by pathogenic nsSNPs in TEN1 protein. We have superimposed the six pathogenic mutants (W13G, L26P, G77R, R92H, R92C, and C96Y) of TEN1 protein onto the structure of WT protein using PyMol (Figure 7A–F). Mutation G77R in the β4-strand of TEN1 protein showed a remarkable conformational change with the highest RMSD values in comparison with other mutations (Figure 7C). R92H and R92C mutations are involving the substitution of arginine by a small histidine and cysteine, thus expecting to affect the conformation of TEN1 protein which is evident from changes in RMSD values of backbone atoms (Figure 7D,E). Other three pathogenic mutations (W13G, L26P, and C96Y) are also showing a considerable structural change in the local structure as compared to WT.

### Aggregation propensities analysis

Protein solubility is one of the critical attribute primarily related to its function [64,65]. Insoluble parts in proteins often tend to form an aggregate which leads to development of many diseases including, amyloidoses [66], Alzheimer’s [67], and Parkinson diseases [68]. Aggregation propensity analysis was performed in the context of identification of a disease or pathogenic SNPs. SODA classifies SNPs based on changes in α-helix and β-strand propensities; aggregation and disorder score, etc. Out of eight pathogenic mutations obtained from MutPred and PhD-SNP tools, six (75%) were found to have an increased tendency to form an aggregate (Table 2). These aggregate forming potential of amino acid residues are primarily located at the C-terminal of TEN1 protein. Replacement of Arg92 by cysteine or histidine is considerably more prone to form an aggregate in comparison with other pathogenic mutations.

**Table 2:** Predicted aggregation scores of wild-type and mutant TEN1 proteins using SODA server

Variants	Helix	Strand	Aggregation	Disorder	SODA	Remark
4JOI*	0.293	0.316	−4.44	0.089		
W13G	−0.211	−0.75	4.7	0.748	4.072	More soluble
L26P	−1.086	−0.695	8.87	0.339	8.18	More soluble
C58Y	−0.139	0.259	−10.084	−0.059	−8.416	Less soluble
G70A	5.76	−4.364	−12.264	0.021	−10.323	Less soluble
G77R	7.374	−5.892	−6.704	0.166	−1.741	Less soluble
R92H	0.768	−0.72	−15.677	−0.041	−16.19	Less soluble
R92C	1.575	−1.358	−42.972	0.114	−45.157	Less soluble
C96Y	1.861	−1.429	−8.643	0.067	−6.102	Less soluble

4JOI* = PDB ID of wild-type

### Structural and functional consequence of mutations

The OB-fold of TEN1 comprises of five antiparallel β-strands folded into a complex β-barrel flanked by two α-helices. N-terminal residues forming a long coil and plays a crucial role in STN1-TEN1 complex formation. Following N-terminal coil, there is a short α-helix (α1) located at an interface of two β-sheets known to provide stability to the structure. However, the C-terminal α-helix (α2) is situated at the opposite end of the β-barrel and spans the whole length of the structure. The N-terminal of STN1 forms a stable heterodimer complex with TEN1. Complex formation between these two proteins is mediated by extensive interactions between the α2- and α3-helices of TEN1 and STN1, respectively (Supplementary Figure S2A). In addition to α-helices, β-barrels of TEN1 and STN1 also form extensive contacts (Supplementary Figure S2B) [31].

Some important amino acid residues, including Val159, Trp160, Ile164, Met167, and Leu168, of α3-helix and some region of flanking coils of STN1 form extensive hydrophobic contacts with the amino acid residues, Met100, Leu104, Leu105, and Ile109, of α2 of TEN1 (Supplementary Figure S2C). Additional interactions between the STN1 and TEN1 are mainly mediated by the conserved Tyr115 of TEN1 α2. Tyr115 is found at the interface of the STN1 and TEN1 and known to form extensive hydrophobic contacts with the side chains of Tyr49, Pro171, and Tyr174 of STN1. Similarly, interactions between the STN1 and TEN1 involve the surface of the β-barrels and the N-terminal tail of TEN1, that runs along the interface of the two domains and form extensive contacts with both of these two proteins (Supplementary Figure S2D). In particular, Arg27 of β1-strand and Arg119 of α2 of TEN1 make an important salt bridge with Asp78 of β2-strand and Asp33 of α2 of STN1, respectively. Further, Met167 of STN1 spans toward α2 and β-barrel interface of TEN1 and form extensive interactions with Leu105, Ala108, and Ile109 of α2 and Tyr9 of the N-terminal coil. It is fascinating that the STN1-TEN1 complex positions the ligand-binding pockets of each subunit on the same side of the heterodimer, forming an extensive ligand-binding pocket [31].

Mutations in protein are often coupled with destabilization or some time associated with disease pathogenesis. Previous studies on mutational analysis demonstrated that the effects of mutations on the stability of protein are primarily owing to changes in hydrophobic contacts [69–71]. However, subsequent studies in a number of cases revealed that substitutions of a large amino acid with smaller ones are usually accompanied by the formation of cavity and effect residue depth and solvent accessibility [72–75]. To find out the impact of a particular mutation on the local and global environment of TEN1 protein structure, we have calculated van der Waals, hydrogen bonding, electrostatic and hydrophobic interactions in WT, and mutant TEN1 using Arpeggio web server (Table 3) [76]. We have estimated the change in the RSA, OSP, and residue depth of wild-type and mutant TEN1 proteins (Figure S3).

**Table 3:** Predictions of non-covalent interactions in wild-type and mutant TEN1 proteins using Arpeggio web server

Variants	van der Waals interactions	Hydrogen bonds	Ionic interactions	Aromatic contacts	Hydrophobic contacts
4JOI*	64	101	18	27	235
P4L	64	102	18	27	235
W13G	67	102	18	7	218
E14D	64	101	13	27	236
V15F	63	102	18	27	263
G23E	63	102	18	27	235
L26P	63	101	18	27	231
C58Y	64	101	18	27	242
L61W	64	103	18	27	240
P64T	64	102	18	27	235
G70A	64	102	18	27	235
G70S	64	102	18	27	235
Y73C	65	101	18	27	222
G77R	65	102	18	27	239
A91V	65	102	18	27	246
R92H	65	100	14	27	235
R92C	64	100	12	27	235
V93M	64	102	18	27	234
C96Y	65	101	18	35	237
C96F	65	101	18	28	243
V97M	64	102	18	27	240

4JOI* = PDB ID of wild-type TEN1 protein.

**Figure 8:**
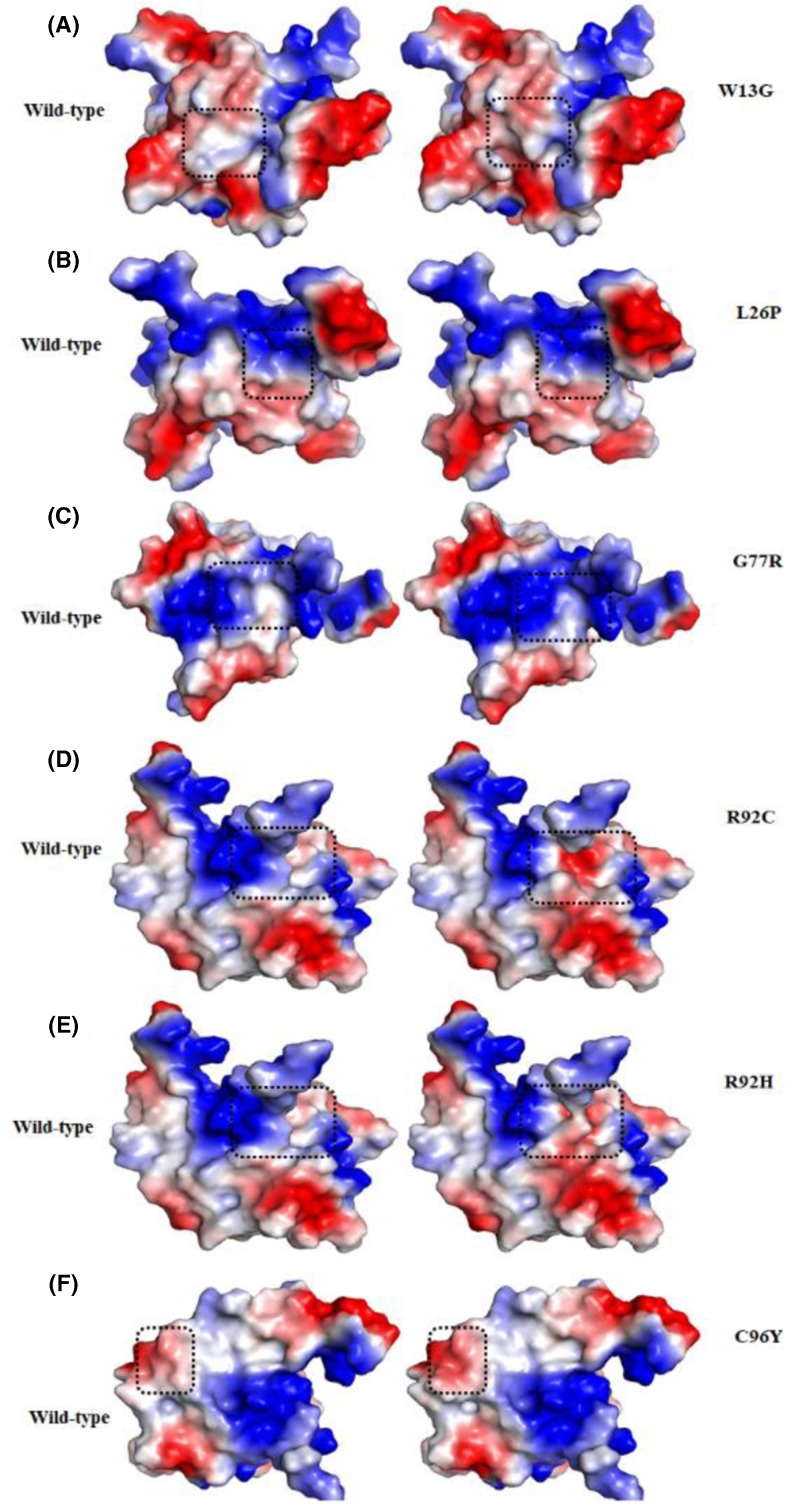
Surface potential representations of WT (left panel) and mutant (right panel) TEN1 proteins **(A)** W13G, (**B)** L26P, **(C)** G77R, **(D)** R92C, **(E)** R92H and **(F)** C96Y. The color ramp for the electrostatic surface potential ranges from blue (most positive) to red (most negative). Surface potential of WT and mutant residues are highlighted by dashed square.

Trp13 is a highly conserved and a buried residue of the N-terminal flanking coil and plays important role in STN1-TEN1 complex formation. Substitutions of larger bulky and highly hydrophobic Trp13 by small, less hydrophobic glycine does not change van der Waals and hydrogen bond interactions significantly, while a large decrease in stacking and hydrophobic interactions are observed (Table 3). Differences in the size and polarity of Trp and glycine affecting the RSA, OSP, and residue depth of protein. Increased RSA value in the Trp13Gly substitution suggested that the substituted residue at Trp13 becomes more accessible to solvent, which is further supported by a decrease in packing density (Supplementary Figure S3). The Surface potential analysis shows a decrease in hydrophobicity in Trp13Gly substitution (Figure 8A). The results suggested that the substitution of Trp13 with the glycine seems indispensable for the stability of TEN1 structure.

Similarly, Leu26 is a highly conserved and buried residue found at the β1-strand of TEN1. Substitutions of hydrophobic Leu26 by a less hydrophobic proline effects only van der Waals and hydrophobic interaction at a little extent (Table 1). However, no significant change was observed for RSA, OSP, surface potential, and residue depth by Leu26Pro mutation (Figure 8B). We may conclude that the incorporation of imino group as a side chain of proline may interfere with the folding pathway of TEN1 without effecting non-covalent interactions.

Gly77 is located in the β4-strand of TEN1 and plays an important role in maintaining the structure and stability (Figure 7D). Substitution of small, hydrophobic, highly conserved, exposed, and functional Gly77 by a large and least hydrophobic, positively charged arginine shows an increase in the van der Waals, hydrogen bonding, and hydrophobic interactions. In consistence, Gly77Arg mutation shows an increase in RSA, a subtle decrease in OSP and residue depth. Gly77Arg mutation increases positively charge environment in the vicinity of Gly77 (Figure 8C). Lethality of Gly77Arg mutation is associated with the changes in RSA of surrounding residues which are critical to maintaining the TEN1 stability.

Arg92 is belonging to the β4-strand of TEN1 and is important for the stability. Substitution of large, highly hydrophilic, conserved, exposed, and functional Arg92 by a small less hydrophilic, positively charged (histidine), and uncharged (cysteine) shows a disruption of one hydrogen bond and a large decrease in the ionic interactions. While, no significant change was observed in the van der Waals, stacking and hydrophobic interactions. Similarly, Arg92His and Arg92Cys mutations show an increase in RSA, and a slight decrease in the OSP and residue depth. The increase in RSA suggesting that the substitution of Arg92 may increase the solvent accessibility of newly incorporated residues. A marked change in surface potential has been observed in Arg92His and Arg92Cys mutations (Figure 8D,E). These results indicate that the lethal effect of Arg92His and Arg92Cys mutations is primarily associated with the changes in hydrogen bonding, ionic interactions, and RSA and thus protein stability.

Cys96 is situated in the β5-strand of TEN1. Substitution of small, less hydrophobic, highly conserved and buried Cys96 by a large and more hydrophobic tyrosine show an increase in the stacking and hydrophobic interactions, while no change was observed in other interactions. Cys96Tyr mutation shows an increase in the RSA and decrease in OSP. No significant change in surface potential has been found except an increase in the hydrophobicity (Figure 8F). Our findings suggest that Cys96Tyr mutation may increase the important hydrophobic and stacking interactions which are being considered as a driving force for protein stability. These increase in stability possibly overcome due to disruptions some important interaction Cys96.

## Conclusion

SNPs are considered as one of the most recurring genetic variants associated with a number of diseases. In the present study, we have examined the consequences of nsSNPs in *TEN1* gene using advanced integrated bioinformatics approach. We have identified a large number of deleterious and destabilizing nsSNPs, which are scattered in different secondary structural components of TEN1 with a high chance of occurring in α1-helix and β5-strands. Aggregation propensity analysis of pathogenic mutation shows that 75% of pathogenic mutations in TEN1 have a tendency to form aggregate and located at C-terminal of TEN1. In-depth structural analysis of these mutations reveals that the pathogenicity of these mutations may be driven through a large structural changes caused by loss/gain of non-covalent intramolecular interactions. The present study provides a mechanistic insight into the understanding of pathogenic mutations in *TEN1* gene and their possible consequences.

## Supporting information

**Supplementary Figure S1:** 

**Supplementary Figure S2:** 

**Supplementary Figure S3:** 

**Supplemental Table S1:** Sequence-based predictions of deleterious nsSNPs in *TEN1* gene using SIFT, PolyPhen-2 and PROVEAN.

**Supplemental Table S2:** Structure-based predictions of destabilizing nsSNPs in *TEN1* gene using STRUM, mCSM, SDM2, DUET and PopMuSic.

## Abbreviations

CC1: conserved telomere maintenance component 1
ESST: environment-specific substitution table
OSP: occluded packing density
PDB: protein data bank
PolyPhen-2: polymorphism phenotyping-2
PROVEAN: protein variation effect analyzer
PSIC: position-specific independent count
RMSD: root mean square deviation
RSA: relative side-chain solvent accessibility
SDM2: site direct mutator 2
SIFT: sorting intolerant from tolerant
SODA: protein solubility from disorder and aggregation
SVM: support vector machine
