# Supplementary material for: Structural and functional impact of non-synonymous SNPs in the CST complex subunit TEN1: structural genomics approach
Source: Biosci Rep. 2019 May 15;39(5):BSR20190312. doi: 10.1042/BSR20190312 (PMC6522806; doi:10.1042/BSR20190312)
Supplement: Supplementary file 1 [file BSR-2019-0312_suppS1.pptx]

## Slide 1
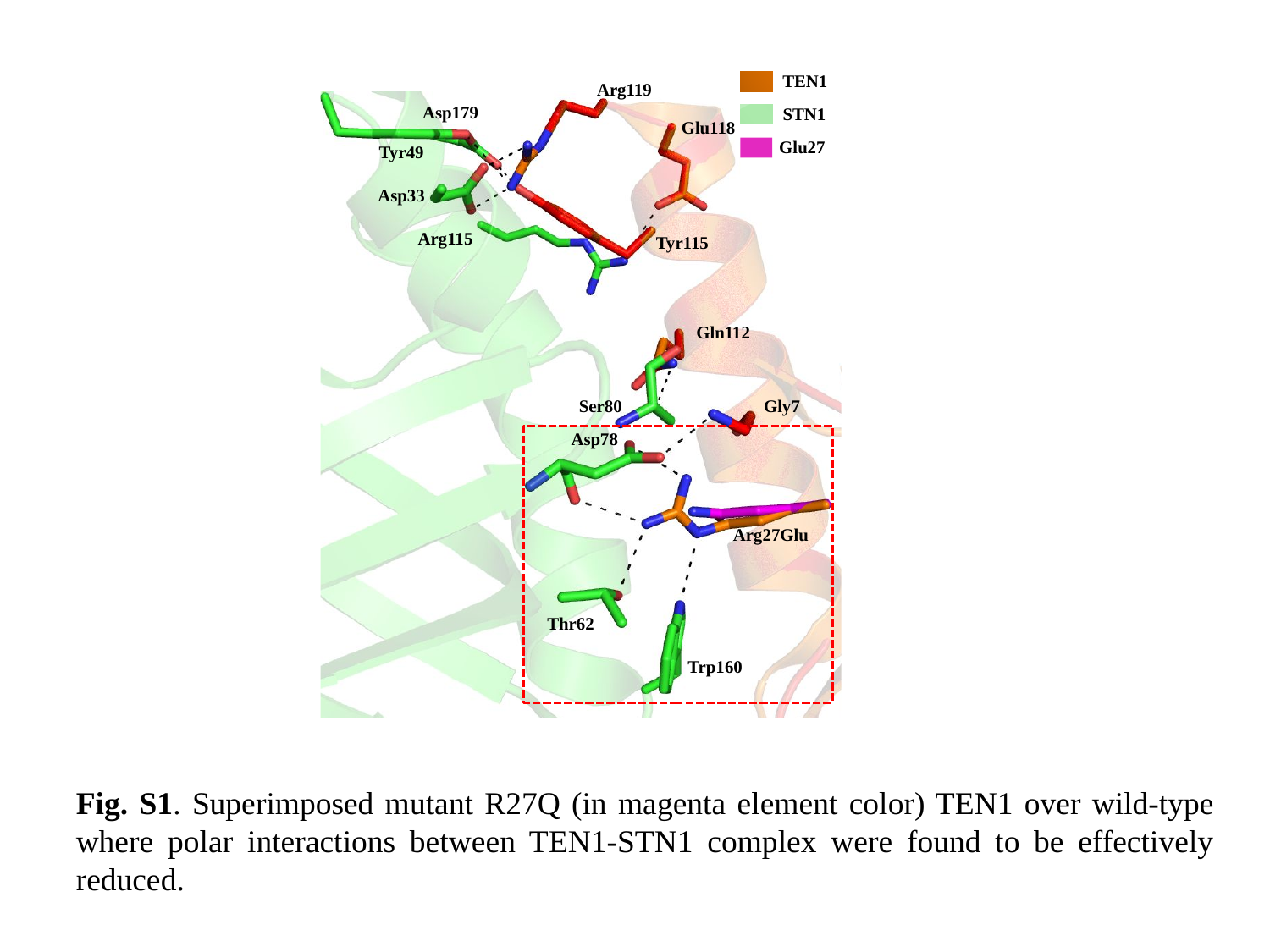

TEN1
Arg119
Asp179
STN1
Glu118
Tyr49
Asp33
Arg115
Tyr115
Gln112
Ser80
Gly7
Asp78
Arg27Glu
Thr62
Trp160
Glu27
Fig. S1. Superimposed mutant R27Q (in magenta element color) TEN1 over wild-type where polar interactions between TEN1-STN1 complex were found to be effectively reduced.
