# Supplementary material for: Structural and functional impact of non-synonymous SNPs in the CST complex subunit TEN1: structural genomics approach
Source: Biosci Rep. 2019 May 15;39(5):BSR20190312. doi: 10.1042/BSR20190312 (PMC6522806; doi:10.1042/BSR20190312)
Supplement: Supplementary file 2 [file BSR-2019-0312_suppS2.pptx]

## Slide 1
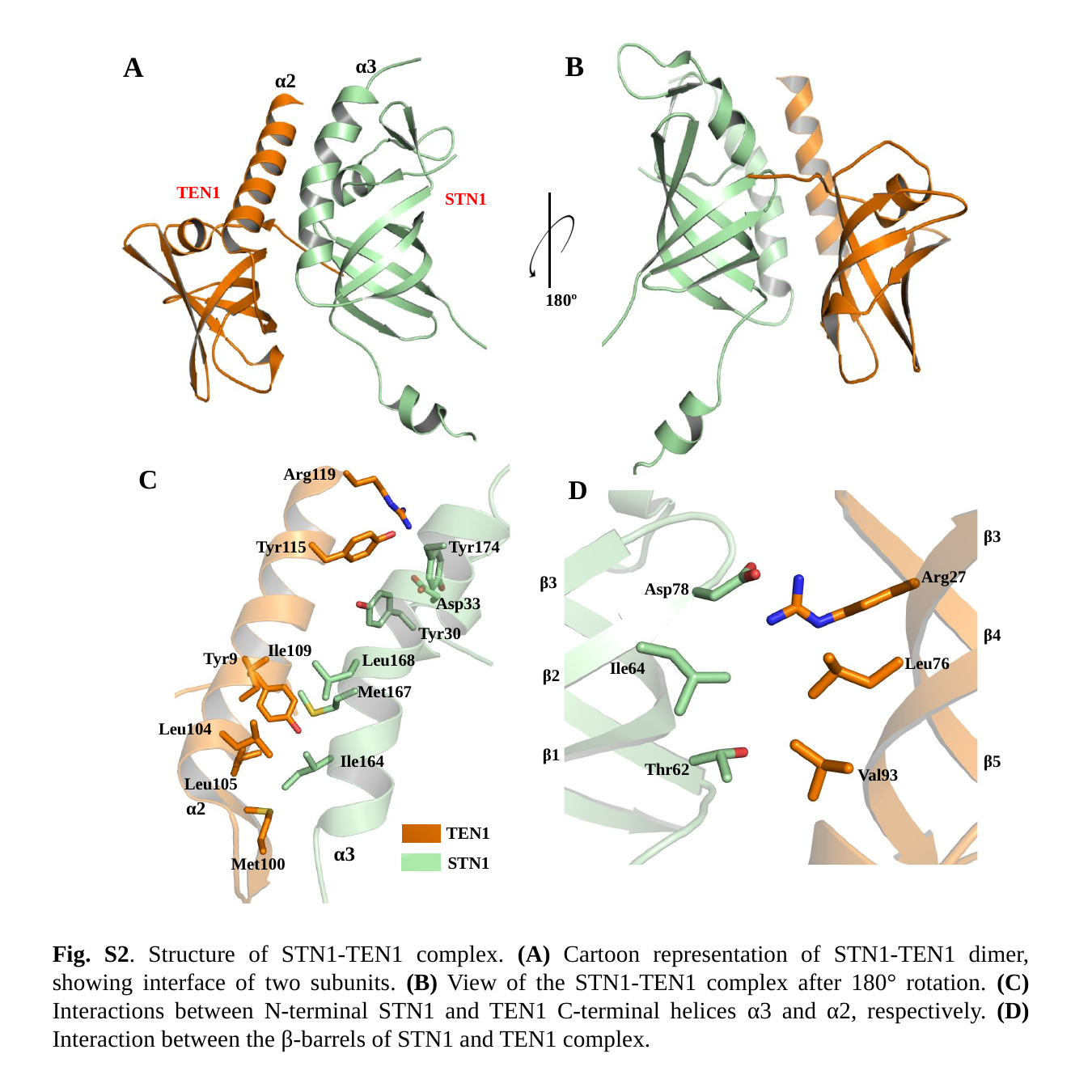

B
A
α3
α2
180º
TEN1
STN1
C
Arg119
Tyr115
Tyr174
Asp33
Tyr30
Ile109
Tyr9
Leu168
Met167
Leu104
Ile164
Leu105
α2
TEN1
α3
STN1
Met100
D
β3
Arg27
β3
Asp78
β4
Leu76
Ile64
β2
β1
β5
Thr62
Val93
Fig. S2. Structure of STN1-TEN1 complex. (A) Cartoon representation of STN1-TEN1 dimer, showing interface of two subunits. (B) View of the STN1-TEN1 complex after 180° rotation. (C) Interactions between N-terminal STN1 and TEN1 C-terminal helices α3 and α2, respectively. (D) Interaction between the β-barrels of STN1 and TEN1 complex.
