# Supplementary material for: Structural and functional impact of non-synonymous SNPs in the CST complex subunit TEN1: structural genomics approach
Source: Biosci Rep. 2019 May 15;39(5):BSR20190312. doi: 10.1042/BSR20190312 (PMC6522806; doi:10.1042/BSR20190312)
Supplement: Supplementary file 3 [file BSR-2019-0312_suppS3.pptx]

## Slide 1
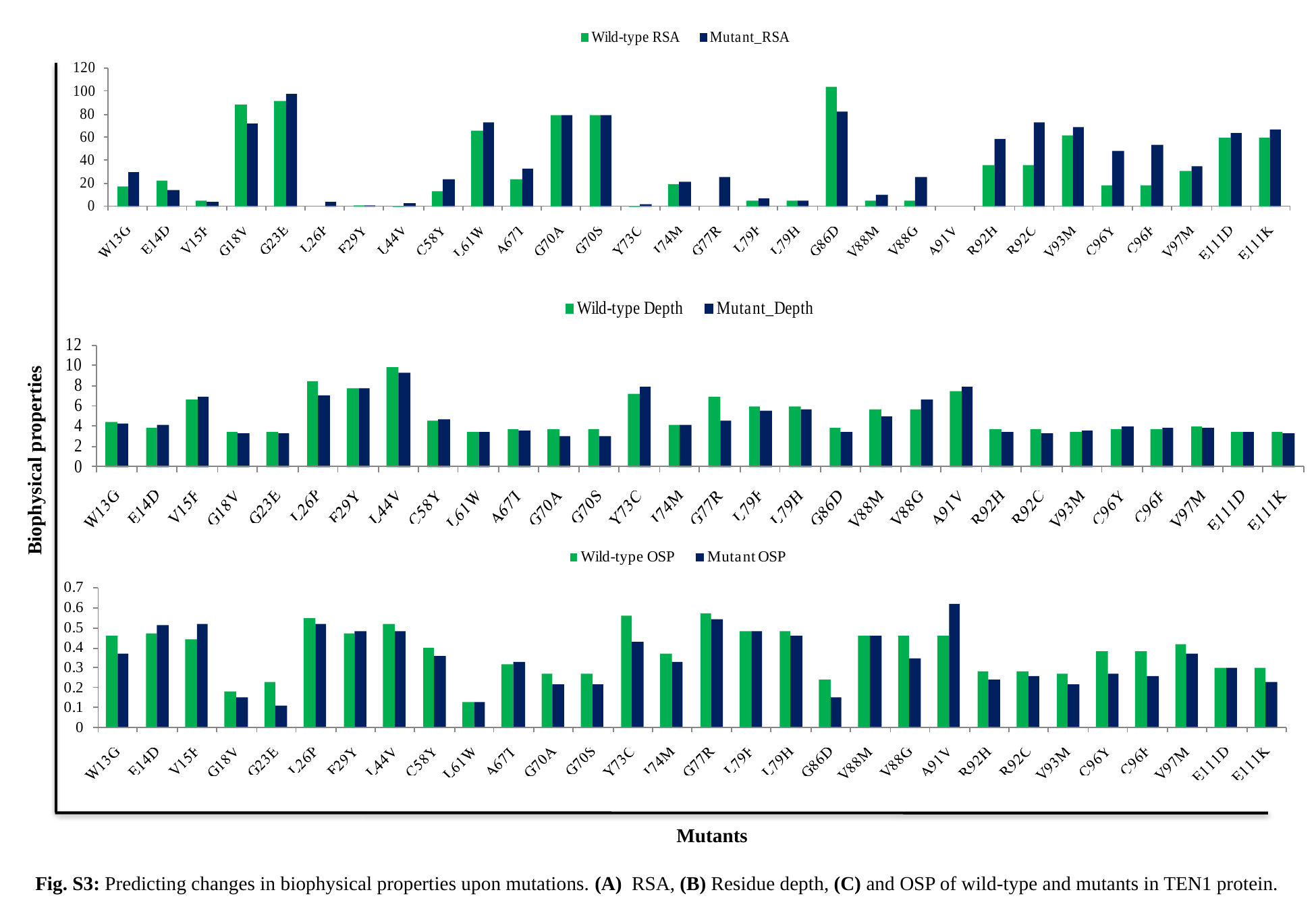

Biophysical properties
Mutants
Fig. S3: Predicting changes in biophysical properties upon mutations. (A) RSA, (B) Residue depth, (C) and OSP of wild-type and mutants in TEN1 protein.
