# Supplementary material for: Structural and functional impact of non-synonymous SNPs in the CST complex subunit TEN1: structural genomics approach
Source: Biosci Rep. 2019 May 15;39(5):BSR20190312. doi: 10.1042/BSR20190312 (PMC6522806; doi:10.1042/BSR20190312)
Supplement: Supplementary file 4 [file BSR-2019-0312_suppS4.pdf]

Table S1: Sequence-based predictions of deleterious nsSNPs in *TEN1* gene using SIFT, PolyPhen-2 and PROVEAN.

S. No.	Variant ID	Variants	Sift_class	SIFT	Polyphen2_class	PolyPhen-2	PROVEAN Score	Remark
1.	rs1322628164	M2V	Deleterious	0	benign	0.018	-2.590	Deleterious
2.	rs892524367	P4L	Deleterious	0	probably damaging	1	-6.734	Deleterious
3.	rs989236496	P6A	Tolerated	0.64	benign	0.21	-0.289	Neutral
4.	rs1212831970	Y9C	Deleterious	0	probably damaging	0.959	-5.335	Deleterious
5.	rs1247295822	P12L	Tolerated	0.35	probably damaging	1	-5.470	Deleterious
6.	rs1333358260	W13G	Deleterious	0.01	probably damaging	1	-8.140	Deleterious
7.	rs1224481693	E14D	Deleterious	0.01	probably damaging	0.999	-2.043	Neutral
8.	rs1175908725	V15 F	Deleterious	0	possibly damaging	0.816	-3.150	Deleterious
9.	rs759172427	A17V	Tolerated	0.07	possibly damaging	0.786	-1.731	Neutral
10.	rs1328038606	G18V	Deleterious	0.04	probably damaging	0.988	-4.522	Deleterious
11.	rs964588646	G23E	Deleterious	0	probably damaging	1	-6.549	Deleterious
12.	rs376979590	T25M	Deleterious	0	possibly damaging	0.855	-3.804	Deleterious
13.	rs1262136645	L26P	Deleterious	0	probably damaging	0.985	-5.011	Deleterious
14.	rs1392752852	T28I	Deleterious	0	probably damaging	1	-3.813	Deleterious
15.	rs1485869353	F29Y	Tolerated	0.11	benign	0.149	-1.914	Neutral
16.	rs1223059981	D36N	Deleterious	0.04	benign	0.386	-3.702	Deleterious
17.	rs1250997925	R41S	Deleterious	0.04	benign	0.099	-2.684	Deleterious
18.	rs1481928740	V42L	Deleterious	0.04	benign	0.05	-1.596	Neutral
19.	rs1178755431	L44V	Deleterious	0.02	possibly damaging	0.652	-1.737	Neutral
20.	rs1251492109	M45V	Tolerated	0.14	benign	0	0.371	Neutral
21.	rs1268278855	M45T	Tolerated	1	benign	0	1.648	Neutral
22.	rs11544990	A46S	Tolerated	0.15	benign	0.444	-1.209	Neutral
23.	rs750169662	H48Y	Deleterious	0.03	benign	0.029	-3.298	Deleterious
24.	rs1425396642	G49R	Tolerated	0.3	benign	0.062	-1.167	Neutral
25.	rs1055013538	D51N	Tolerated	0.44	benign	0.005	-1.520	Neutral
26.	rs1412009927	C58Y	Tolerated	0.17	probably damaging	0.978	-4.679	Deleterious
27.	rs1373602232	T59I	Deleterious	0	probably damaging	0.972	-5.579	Deleterious
28.	rs1360895135	L61F	Tolerated	0.64	possibly damaging	0.654	-1.070	Neutral
29.	rs977512123	L61M	Deleterious	0	probably damaging	0.995	-1.298	Neutral
30.	rs1032051988	L61W	Deleterious	0.01	probably damaging	0.997	-3.746	Deleterious
31.	rs889310547	P64T	Deleterious	0	probably damaging	1	-7.018	Deleterious
32.	rs543068875	H66R	Deleterious	0.03	benign	0	-0.298	Neutral
33.	rs998232737	A67T	Tolerated	0.07	possibly damaging	0.896	-2.281	Neutral

34.	rs998232737	A67S	Tolerated	0.1	probably damaging	0.955	-1.368	Neutral
35.	rs200875806	Q68H	Tolerated	0.38	benign	0.03	-0.004	Neutral
36.	rs975857712	V69M	Tolerated	0.16	benign	0.034	-1.133	Neutral
37.	rs951187486	G70A	Deleterious	0	probably damaging	0.92	-4.795	Deleterious
38.	rs1180274799	G70S	Deleterious	0	possibly damaging	0.89	-4.344	Deleterious
39.	rs1445270614	Y73C	Deleterious	0	probably damaging	0.991	-8.386	Deleterious
40.	rs374268204	I74M	Tolerated	0.74	benign	0.028	0.896	Neutral
41.	rs370881785	I74V	Tolerated	0.37	benign	0.001	-0.129	Neutral
42.	rs929018097	V75I	Deleterious	0.02	benign	0.322	-0.561	Neutral
43.	rs1358892195	G77R	Deleterious	0	probably damaging	0.997	-7.789	Deleterious
44.	rs1300674402	E78D	Tolerated	0.06	benign	0.059	-2.486	Neutral
45.	rs1384576801	L79I	Tolerated	0.48	possibly damaging	0.69	-0.754	Neutral
46.	rs1384576801	L79F	Tolerated	0.09	benign	0.162	-1.947	Neutral
47.	rs1452705083	L79H	Tolerated	0.06	benign	0.162	-3.921	Deleterious
48.	rs1322757890	H81R	Tolerated	0.46	benign	0.012	-0.861	Neutral
49.	rs1296287868	H81Y	Tolerated	0.59	benign	0.029	-1.593	Neutral
50.	rs760290715	D84N	Tolerated	0.2	benign	0.059	-1.909	Neutral
51.	rs777056924	R85G	Tolerated	0.6	benign	0	2.910	Neutral
52.	rs1433184403	G86D	Tolerated	0.17	benign	0.093	-1.061	Neutral
53.	rs201043191	V88M	Tolerated	0.4	benign	0.059	-0.489	Neutral
54.	rs201043191	V88L	Tolerated	0.33	benign	0.02	-0.613	Neutral
55.	rs562062613	V88G	Deleterious	0	possibly damaging	0.549	-5.219	Deleterious
56.	rs1401886733	A91V	Tolerated	0.06	probably damaging	0.983	-3.579	Deleterious
57.	rs1016457057	R92H	Deleterious	0	probably damaging	0.986	-4.789	Deleterious
58.	rs759839415	R92C	Deleterious	0	probably damaging	0.996	-7.316	Deleterious
59.	rs905216603	V93M	Deleterious	0	probably damaging	0.927	-1.895	Neutral
60.	rs1286634889	C96Y	Deleterious	0	probably damaging	0.99	-9.158	Deleterious
61.	rs1286634889	C96F	Deleterious	0	probably damaging	0.979	-9.263	Deleterious
62.	rs368827427	V97M	Deleterious	0.01	probably damaging	0.986	-2.947	Deleterious
63.	rs1012536290	M100L	Tolerated	0.12	benign	0.009	-1.316	Neutral
64.	rs1225289402	N101I	Deleterious	0	probably damaging	0.918	-6.337	Deleterious
65.	rs1270235483	P103L	Tolerated	0.16	benign	0.034	-2.477	Neutral
66.	rs1216398771	E106D	Deleterious	0.02	probably damaging	0.946	-2.474	Neutral
67.	rs761196403	E106Q	Tolerated	0.39	possibly damaging	0.652	-1.316	Neutral
68.	rs765493477	R110Q	Tolerated	1	benign	0.001	0.811	Neutral
69.	rs1230794805	R110W	Deleterious	0.01	possibly damaging	0.663	-2.447	Neutral
70.	rs1158635929	E111G	Deleterious	0.03	benign	0.024	-4.905	Deleterious
71.	rs1412372476	E111D	Tolerated	0.08	possibly damaging	0.547	-2.358	Neutral

72.	rs1411814496	E111K	Tolerated	0.06	benign	0.041	-2.179	Neutral
73.	rs981085448	Y115H	Tolerated	0.07	benign	0.072	-2.825	Deleterious
74.	rs1407328186	Y115F	Tolerated	0.3	possibly damaging	0.907	-1.386	Neutral
75.	rs372525536	K116E	Tolerated	0.21	benign	0.009	0.017	Neutral
76.	rs772974788	R119G	Deleterious	0	possibly damaging	0.806	-4.575	Deleterious
77.	rs772974788	R119W	Deleterious	0	probably damaging	0.988	-6.254	Deleterious
78.	rs377039339	R119Q	Deleterious	0.02	benign	0.263	-2.546	Deleterious

Table S2: Structure-based predictions of destabilizing nsSNPs in *TEN1* gene using STRUM, mCSM, SDM2, DUET and PopMuSic.

S. No.	Variant ID	Variants	STRUM		mCSM		SDM2		DUET		PoPMuSiC	
			Score (Kcal/mol)	Remark	Score (Kcal/mol)	Remark	Score (Kcal/mol)	Remark	Score (Kcal/mol)	Remark	Score (Kcal/mol)	Remark
1.	rs1322628164	M2V	0.07	Stabilizing	0.0	Destabilizing	0.0	Destabilizing	0.102	Stabilizing	-0.349	Stabilizing
2.	rs892524367	P4L	-0.11	Destabilizing	-0.307	Destabilizing	-0.04	Destabilizing	0.007	Stabilizing	0.402	Destabilizing
3.	rs989236496	P6A	0.42	Stabilizing	-0.369	Destabilizing	-0.2	Destabilizing	-0.094	Destabilizing	0.888	Destabilizing
4.	rs1212831970	Y9C	0.58	Stabilizing	-1.12	Destabilizing	0.06	Stabilizing	-0.932	Destabilizing	1.283	Destabilizing
5.	rs1247295822	P12L	0.57	Stabilizing	-0.348	Destabilizing	3.38	Stabilizing	0.677	Stabilizing	-1.417	Stabilizing
6.	rs1333358260	W13G	-1.71	Destabilizing	-3.084	Destabilizing	-0.77	Destabilizing	-2.655	Destabilizing	3.096	Destabilizing
7.	rs1224481693	E14D	-0.15	Destabilizing	-1.146	Destabilizing	-0.58	Destabilizing	-0.954	Destabilizing	1.620	Destabilizing
8.	rs1175908725	V15 F	-0.42	Destabilizing	-1.421	Destabilizing	-1.19	Destabilizing	-1.626	Destabilizing	1.076	Destabilizing
9.	rs759172427	A17V	0.24	Stabilizing	-0.205	Destabilizing	-0.95	Destabilizing	-0.169	Destabilizing	0.498	Destabilizing
10.	rs1328038606	G18V	-0.6	Destabilizing	-0.357	Destabilizing	-1.6	Destabilizing	-0.527	Destabilizing	1.123	Destabilizing
11.	rs964588646	G23E	-0.43	Destabilizing	-0.938	Destabilizing	-2.58	Destabilizing	-1.266	Destabilizing	0.372	Destabilizing
12.	rs376979590	T25M	0.35	Stabilizing	-0.005	Destabilizing	-0.78	Destabilizing	-0.086	Destabilizing	0.414	Destabilizing
13.	rs1262136645	L26P	-2.9	Destabilizing	-1.923	Destabilizing	-4.31	Destabilizing	-2.62	Destabilizing	3.266	Destabilizing
14.	rs1392752852	T28I	0.1	Stabilizing	0.397	Stabilizing	1.81	Stabilizing	1.113	Stabilizing	-0.114	Stabilizing
15.	rs1485869353	F29Y	-0.42	Destabilizing	-0.676	Destabilizing	-0.76	Destabilizing	-0.467	Destabilizing	1.553	Destabilizing
16.	rs1223059981	D36N	0.26	Stabilizing	-1.046	Destabilizing	-0.18	Destabilizing	-0.748	Destabilizing	0.442	Destabilizing
17.	rs1250997925	R41S	0.38	Stabilizing	-1.773	Destabilizing	-1.32	Destabilizing	-1.893	Destabilizing	1.910	Destabilizing
18.	rs1481928740	V42L	-0.09	Destabilizing	-0.165	Destabilizing	-1.5	Destabilizing	-0.104	Destabilizing	1.053	Destabilizing
19.	rs1178755431	L44V	-1.07	Destabilizing	-1.734	Destabilizing	-1.58	Destabilizing	-1.87	Destabilizing	1.519	Destabilizing
20.	rs1251492109	M45V	1.28	Stabilizing	-1.02	Destabilizing	1.39	Stabilizing	-0.164	Destabilizing	0.786	Destabilizing
21.	rs1268278855	M45T	1.25	Stabilizing	-0.897	Destabilizing	0.02	Stabilizing	-0.283	Destabilizing	1.073	Destabilizing
22.	rs11544990	A46S	0.26	Stabilizing	-1.843	Destabilizing	-2.47	Destabilizing	-1.974	Destabilizing	1.151	Destabilizing
23.	rs750169662	H48Y	0.12	Stabilizing	1.401	Stabilizing	-0.16	Destabilizing	1.343	Stabilizing	-0.365	Stabilizing
24.	rs1425396642	G49R	0.39	Stabilizing	-0.176	Destabilizing	-0.68	Destabilizing	-0.023	Destabilizing	-0.100	Stabilizing
25.	rs1055013538	D51N	0.53	Stabilizing	0.107	Stabilizing	0.06	Stabilizing	0.336	Stabilizing	0.555	Destabilizing
26.	rs1412009927	C58Y	-1.76	Destabilizing	-0.933	Destabilizing	-1.60	Destabilizing	-1.099	Destabilizing	1.219	Destabilizing
27.	rs1373602232	T59I	0.22	Stabilizing	0.073	Stabilizing	0.61	Stabilizing	0.435	Stabilizing	0.294	Destabilizing
28.	rs1360895135	L61F	-0.93	Destabilizing	-0.587	Destabilizing	-0.05	Destabilizing	-0.598	Destabilizing	-0.041	Stabilizing
29.	rs977512123	L61M	-0.64	Destabilizing	-0.471	Destabilizing	-0.48	Destabilizing	-0.538	Destabilizing	-0.200	Stabilizing
30.	rs1032051988	L61W	-0.8	Destabilizing	-0.597	Destabilizing	-0.43	Destabilizing	-0.717	Destabilizing	0.091	Destabilizing
31.	rs889310547	P64T	0.51	Stabilizing	-0.718	Destabilizing	-0.38	Destabilizing	-0.437	Destabilizing	0.105	Destabilizing
32.	rs543068875	H66R	0.44	Stabilizing	-0.384	Destabilizing	-0.11	Destabilizing	-0.167	Destabilizing	-0.375	Stabilizing
33.	rs998232737	A67T	-0.08	Destabilizing	-0.941	Destabilizing	-0.62	Destabilizing	-0.724	Destabilizing	0.494	Destabilizing
34.	rs998232737	A67S	0.28	Stabilizing	-1.142	Destabilizing	-1.38	Destabilizing	-1.087	Destabilizing	0.792	Destabilizing
35.	rs200875806	Q68H	0.4	Stabilizing	-0.579	Destabilizing	1.08	Stabilizing	-0.294	Destabilizing	-0.087	Stabilizing
36.	rs975857712	V69M	0.0	Stabilizing	-0.332	Destabilizing	-0.45	Destabilizing	-0.38	Destabilizing	-0.083	Stabilizing
37.	rs951187486	G70A	-0.7	Destabilizing	-0.623	Destabilizing	-2.71	Destabilizing	-1.083	Destabilizing	0.500	Destabilizing
38.	rs1180274799	G70S	-0.83	Destabilizing	-1.007	Destabilizing	-4.3	Destabilizing	-1.596	Destabilizing	0.812	Destabilizing
39.	rs1445270614	Y73C	0.3	Stabilizing	-1.875	Destabilizing	-1.42	Destabilizing	-2.004	Destabilizing	2.591	Destabilizing
40.	rs374268204	I74M	-0.4	Destabilizing	-0.739	Destabilizing	-0.97	Destabilizing	-0.765	Destabilizing	1.326	Destabilizing
41.	rs370881785	I74V	0.3	Stabilizing	-1.304	Destabilizing	0.29	Stabilizing	-0.885	Destabilizing	1.177	Destabilizing
42.	rs929018097	V75I	0.16	Stabilizing	-0.683	Destabilizing	-0.6	Destabilizing	-0.438	Destabilizing	0.460	Destabilizing

43.	rs1358892195	G77R	-1.35	Destabilizing	-1.22	Destabilizing	-2.73	Destabilizing	-1.215	Destabilizing	1.717	Destabilizing
44.	rs1300674402	E78D	-0.0	Destabilizing	-1.248	Destabilizing	-1.06	Destabilizing	-1.188	Destabilizing	0.449	Destabilizing
45.	rs1384576801	L79I	-0.29	Destabilizing	-1.207	Destabilizing	0.77	Stabilizing	-0.652	Destabilizing	0.719	Destabilizing
46.	rs1384576801	L79F	-0.52	Destabilizing	-1.672	Destabilizing	-0.66	Destabilizing	-1.634	Destabilizing	1.380	Destabilizing
47.	rs1452705083	L79H	-1.74	Destabilizing	-1.851	Destabilizing	-1.44	Destabilizing	-1.883	Destabilizing	3.088	Destabilizing
48.	rs1322757890	H81R	0.65	Stabilizing	0.004	Stabilizing	-0.24	Destabilizing	0.201	Stabilizing	0.249	Destabilizing
49.	rs1296287868	H81Y	0.56	Stabilizing	0.78	Stabilizing	-0.16	Destabilizing	0.834	Stabilizing	0.210	Destabilizing
50.	rs760290715	D84N	0.01	Stabilizing	0.072	Destabilizing	0.02	Destabilizing	0.188	Destabilizing	-0.186	Stabilizing
51.	rs777056924	R85G	1.45	Stabilizing	-0.136	Destabilizing	2.6	Stabilizing	0.524	Stabilizing	-0.113	Stabilizing
52.	rs1433184403	G86D	-0.26	Destabilizing	-0.813	Destabilizing	-3.66	Destabilizing	-1.207	Destabilizing	0.809	Destabilizing
53.	rs201043191	V88M	-0.67	Destabilizing	-1.029	Destabilizing	-2.17	Destabilizing	-1.398	Destabilizing	1.783	Destabilizing
54.	rs201043191	V88L	0.11	Stabilizing	-1.011	Destabilizing	-1.54	Destabilizing	-1.048	Destabilizing	1.026	Destabilizing
55.	rs562062613	V88G	-1.59	Destabilizing	-2.268	Destabilizing	-3.2	Destabilizing	-2.813	Destabilizing	4.095	Destabilizing
56.	rs1401886733	A91V	-0.86	Destabilizing	-0.638	Destabilizing	0.95	Destabilizing	-0.005	Destabilizing	0.415	Destabilizing
57.	rs1016457057	R92H	-0.45	Destabilizing	-0.965	Destabilizing	0.28	Stabilizing	-0.722	Destabilizing	0.166	Destabilizing
58.	rs759839415	R92C	-0.14	Destabilizing	-0.52	Destabilizing	0.02	Stabilizing	-0.368	Destabilizing	0.248	Destabilizing
59.	rs905216603	V93M	-0.43	Destabilizing	-0.462	Destabilizing	-1.19	Destabilizing	-0.518	Destabilizing	0.599	Destabilizing
60.	rs1286634889	C96Y	-1.25	Destabilizing	-1.157	Destabilizing	-0.51	Destabilizing	-0.963	Destabilizing	0.905	Destabilizing
61.	rs1286634889	C96F	-0.67	Destabilizing	-1.125	Destabilizing	-0.5	Destabilizing	-0.975	Destabilizing	0.201	Destabilizing
62.	rs368827427	V97M	-0.11	Destabilizing	-0.325	Destabilizing	-0.26	Destabilizing	-0.24	Destabilizing	0.367	Destabilizing
63.	rs1012536290	M100L	-0.06	Destabilizing	-0.518	Destabilizing	0.39	Stabilizing	-0.104	Destabilizing	0.749	Destabilizing
64.	rs1225289402	N101I	-0.12	Destabilizing	0.197	Stabilizing	0.55	Stabilizing	0.485	Stabilizing	0.704	Stabilizing
65.	rs1270235483	P103L	0.47	Stabilizing	-0.43	Destabilizing	1.57	Stabilizing	0.32	Stabilizing	-0.452	Stabilizing
66.	rs1216398771	E106D	0.23	Stabilizing	-1.132	Destabilizing	-0.86	Destabilizing	-1.013	Destabilizing	1.312	Destabilizing
67.	rs761196403	E106Q	0.6	Stabilizing	-0.148	Destabilizing	-0.69	Destabilizing	0.076	Stabilizing	0.273	Destabilizing
68.	rs765493477	R110Q	0.13	Stabilizing	-0.336	Destabilizing	-0.10	Destabilizing	-0.062	Destabilizing	-0.115	Stabilizing
69.	rs1230794805	R110W	-0.09	Destabilizing	-0.278	Destabilizing	-0.32	Destabilizing	-0.491	Destabilizing	0.439	Destabilizing
70.	rs1158635929	E111G	0.57	Stabilizing	-1.191	Destabilizing	-1.68	Destabilizing	-1.429	Destabilizing	1.426	Destabilizing
71.	rs1412372476	E111D	-0.19	Destabilizing	-0.998	Destabilizing	-1.13	Destabilizing	-0.935	Destabilizing	0.709	Destabilizing
72.	rs1411814496	E111K	-0.17	Destabilizing	-0.476	Destabilizing	-1.0	Destabilizing	-0.303	Destabilizing	0.497	Destabilizing
73.	rs981085448	Y115H	0.23	Stabilizing	-0.256	Destabilizing	-0.26	Destabilizing	0.089	Stabilizing	-0.331	Stabilizing
74.	rs1407328186	Y115F	0.15	Stabilizing	-0.625	Destabilizing	0.12	Stabilizing	-0.186	Destabilizing	0.332	Destabilizing
75.	rs372525536	K116E	-0.12	Destabilizing	0.114	Stabilizing	0.99	Stabilizing	0.718	Stabilizing	-0.569	Stabilizing
76.	rs772974788	R119G	1.39	Stabilizing	-0.102	Destabilizing	0.0	Destabilizing	-0.074	Destabilizing	-0.202	Stabilizing
77.	rs772974788	R119W	-0.02	Destabilizing	-0.415	Destabilizing	0.0	Destabilizing	-0.586	Destabilizing	-0.040	Stabilizing
78.	rs377039339	R119Q	0.67	Stabilizing	-0.103	Destabilizing	0.0	Destabilizing	0.026	Stabilizing	-0.784	Stabilizing
